# The sustainability paradox of processing plant proteins

**DOI:** 10.1038/s41538-023-00214-1

**Published:** 2023-07-25

**Authors:** Patrícia Duque-Estrada, Iben Lykke Petersen

**Affiliations:** Department of Food Science, Food Analytics and Biotechnology Section, University of Copenhagen, Rolighedsvej 26, 1958 Frederiksberg C, Denmark

**Keywords:** Proteomics, Proteins, Metabolomics

## Abstract

The production of sustainable plant-based foods is not simply a question of which process has the lowest environmental impact in the food chain. We have to consider that different degrees of processing might result in different degrees of plant protein nutritional quality in the final food product.

## Where the paradox stands

Protein transition has been considered one of the approaches to tackle climate change by reducing the intake of animal proteins and increasing the intake of plant proteins^1^. However, this transition is challenging due to the lower nutritional quality of plant proteins compared to animal proteins, limiting their incorporation into our diets. Protein nutritional quality is defined as the ability of a protein to supply enough of the essential amino acids (EAAs), those that can not be synthesized in the human body, according to the dietary requirements of a target group^2^. Therefore, the protein quality is largely affected by EAAs composition as well as protein digestibility, meaning how much of ingested protein is broken down to amino acids and di- or tripeptides in our digestive system and thereby available for absorption^3^.

Unprocessed plant proteins by nature have low protein nutritional quality due to their inability to provide enough of certain EAAs needed for human metabolism and the complexity of their matrix, limiting protein digestibility. In this complex matrix, the presence of cell wall, non-protein components (e.g. fibers and starch), and antinutritional factors (ANFs) (e.g. protease inhibitors, phytates, non-starch polysaccharides, and polyphenols) can decrease protein digestibility and thereby the utilization of ingested proteins^2^. The presence of ANFs and non-protein components in less purified protein ingredients is what partially explains why most of the purified ingredients have higher protein nutritional quality. Thereby, food processing strategies can be applied to improve the nutritional quality of plant protein ingredients by degrading the cell wall, removing non-protein components, and inactivating/reducing ANFs^4^. However, some of the processes commonly used by the food industry are not sustainable and the use of less purified plant protein ingredients has been preferred since it requires fewer processing steps and less use of water and energy. Here we encounter the sustainability paradox which stands for the fact that less processing most often results in lower protein nutritional quality compared to purified protein ingredients that are more processed. But as in all things, it is all a matter of finding the right balance, as we know that although necessary, food processing can simultaneously impact protein´s nutritional quality, both positively and negatively.

Often, processing plant proteins to a certain extent is necessary to be able to digest proteins better and thereby utilize the EAAs. But food processing can also result in negative protein modifications by protein oxidation, protein glycation via Maillard reaction, protein crosslinking, and amino acid racemization^5–7^ that all can alter the protein nutritional quality, depending on the reaction level and process conditions applied. For instance, protein oxidation can result in protein fragmentation and aggregation, and irreversible modification of EAAs. At the same time, the Maillard reaction can lead to the loss of EAAs, and the formation of products such as acrylamide and advanced glycation end-products (AGEs)^8^. Currently, the health effects of AGEs are debatable, both beneficial and detrimental^8^. Therefore, the negative impact of protein modifications on nutritional quality is associated with a decrease in protein digestibility and the impairment of amino acid absorption and utilization by the body^7^. Thereby, more studies are needed to identify and understand the effect of food processing on protein modifications and their effects on protein nutritional quality and consequences on human health.

Overall, the impact of protein modifications on plant protein nutritional quality will depend on the food processing conditions applied to the protein ingredients and final plant-based food products. Considering that plant-based foods products are processed, recently, public health and nutrition scientists have argued that these products are unhealthy since they are considered ultra-processed foods (UPFs). As described by Scrinis and Monteiro^9^, “UPFs are defined as industrial formulations manufactured by deconstructing foods into their component parts, modifying them and recombining them with a myriad of additives and little, if any, whole foods”. Moreover, UPFs are also products meant for convenience, with high palatable and profitability. UPFs are considered unhealthy because most of them are nutritionally unbalanced, with high sugar, sodium, saturated- and trans-fat content, and/or high energy density ^9^. Therefore, the high intake of UPFs has been associated with nutrient inadequacies and deficiency contributing to the development of chronic non-communicable diseases^9^. However, according to this definition, there is no consideration of the impact of food processing on the nutritional quality of the product, but rather a focus on their formulation. The term UPFs has been extensively criticized among the scientific community as being inappropriate, confusing, and misleading^10–12^. We believe that classifying all plant-based foods as UPFs based on their formulation is not ideal since it implies that all these products are nutritionally unbalanced. For instance, Penna Franca et al.^13^ have shown that the energy density of 90% of meat analogs analyzed was adequate or below the recommendations. Meanwhile, 67.5% of the products had food additives which shows that the formulation of meat analogs should be improved to reach a more clean food label. Therefore, we consider that the use of UPFs term to classify plant-based foods should be adjusted to properly categorize these products. Moreover, considering the effect of food processing on protein modifications and their possible health consequences, both the type and level of food processing should be taken into consideration in food classification systems.

**Fig. 1:**
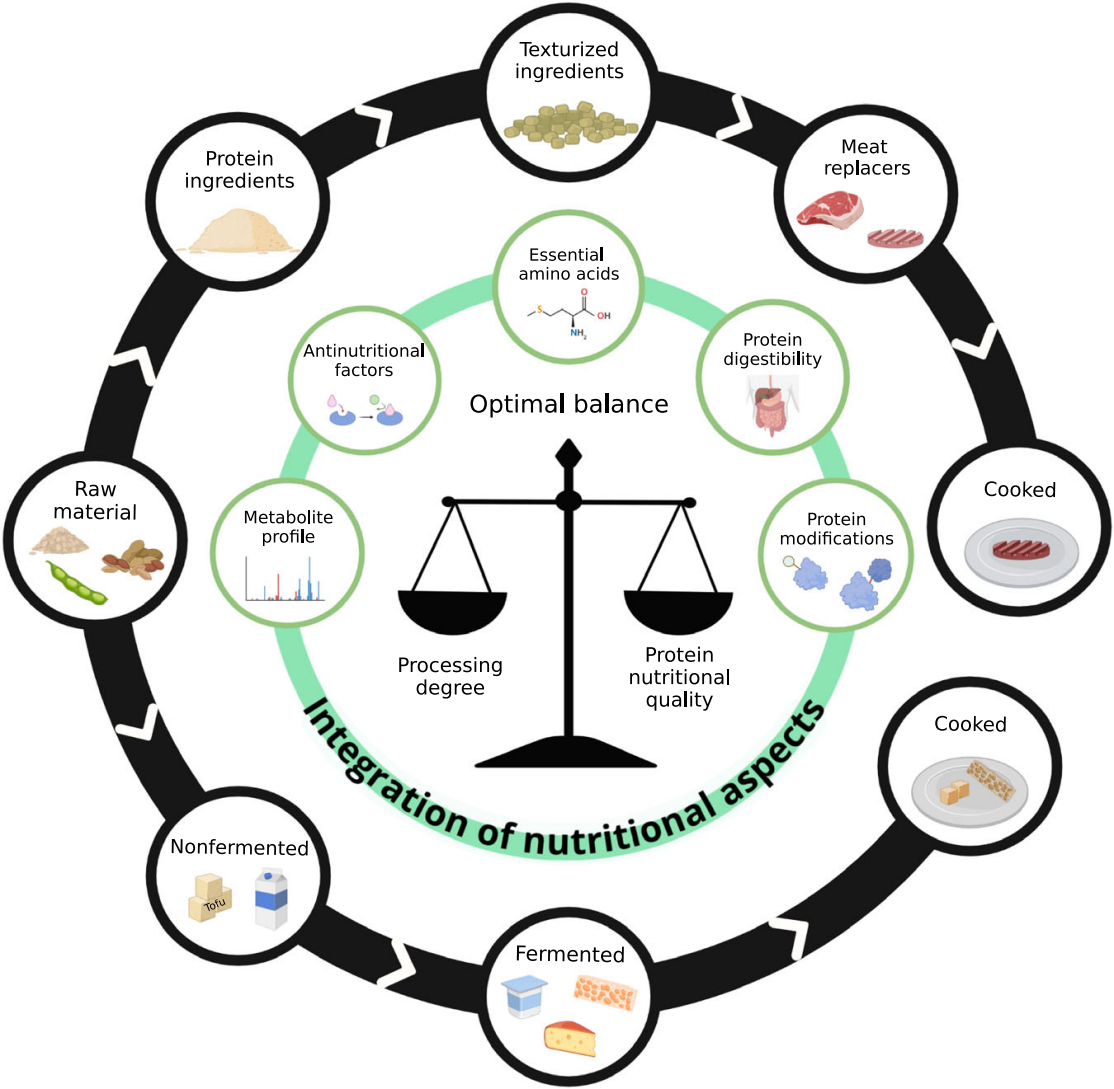
Schematic representation of the integration of nutritional aspects to study the link between the protein nutritional quality and the processing degree of plant proteins aiming to find an optimal balance, considering different steps in the production chain. To access the overall nutritional quality of plant-based foods the digestibility and bioavailability of other nutrients than proteins should be considered. The outer black circle represents a simple example of the production of different plant protein-based foods from raw materials (on the left) to final cooked food (on the right), the direction is represented by the white arrows. Created with BioRender.com.

Considering how intrinsically connected the degree of processing and the nutritional quality of plant proteins are, we suggest that the integration of aspects such as the identification of protein modifications, EAA content, and protein digestibility (Fig. 1) should be taken into consideration when aiming to find an optimal balance between the protein nutritional quality and food processing of plant protein-based foods. In addition, tracking protein nutritional quality during different processing steps should also be considered, since modifications will happen during all these steps of processing from raw material to a final food product. Is important to note that the effect of food processing on the bioavailability of other micro- and macronutrients should be considered to access the overall nutritional quality of plant-based foods. Though this is not part of the scope of this paper in which we will only focus on the protein nutritional quality.

## From raw to cooked food

Lately, research has focused on understanding the impact of food processing on the protein quality of ingredients, e.g. protein concentrates, isolates, or texturized proteins, and less focus on a final food product, let alone the cooked food. To get to a final food product, protein ingredients will go through further processing steps and will be combined with other ingredients for texture and sensory aspects purposes. Processing conditions and the presence of other components in the food matrix will further result in chemical and physical changes, as described earlier, and their impact on protein digestibility or amino acid content is poorly understood in plant proteins and plant-based foods.

Furthermore, cooking (as a final step in the food chain) can also affect protein digestibility due to changes in protein structure, affecting digestion and absorption rate^14^. For instance, it is well known that meat proteins will go through chemical changes during cooking because of their native structure^15^ and that, depending on the cooking temperature and time, protein digestibility might increase^2^. On the contrary, for plant proteins that have already been processed and modified to a certain extent compared to their native protein structure, it is not clear what happens during cooking. A study investigating unprocessed legumes (i.e. beans, lentils, chickpeas) has shown that wet cooking, such as boiling and autoclaving, improves protein digestibility, while dry roasting tends to decrease it^2^. But it is not known whether this also applies to more processed plant protein ingredients.

**Fig. 2:**
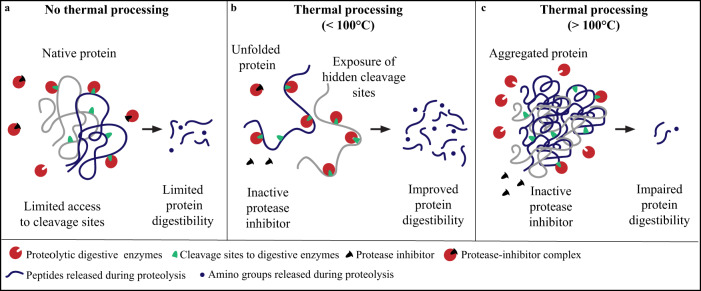
Schematic representation of the effect of thermal processing on plant protein structure and protease inhibitors (antinutritional factor) resulting in different levels of protein digestibility. **a** No thermal processing applied; **b** thermal processing applied at temperatures below 100 °C; and **c** thermal processing applied at temperatures above 100 °C.

Generally, it is expected that heating plant proteins at temperatures below 100 °C will improve protein digestibility by changing the protein structure resulting in the exposure of cleavage sites to digestive enzymes and by the inactivation/decrease of most of the ANFs^2,14^ (Fig. 2b). Conversely, temperatures above 100 °C can result in protein oxidation and aggregation which might reduce the accessibility to digestive enzymes, thereby decreasing the protein digestibility^14^ (Fig. 2c). Besides the temperature, processing time and moisture content will also impact the digestibility of plant proteins^7^. Therefore, the protein nutritional quality of the final food might differ from the raw material and the processed protein ingredients used. It is not clear yet how much the combination of different processing steps and the complexity of the food matrix will affect the total protein nutritional quality in plant-based foods.

## Protein modifications as part of the protein nutritional quality assessment

Over the past years, research has evolved to determine protein modifications in animal proteins, which have contributed to the advance in using analytical tools (e.g. mass spectrometry) to determine specific oxidation and Maillard reaction products^16,17^. The same trend has not evolved for plant protein-based foods, with very little research investigating the impact of these modifications, especially on protein nutritional quality^18^. The identification of protein modifications and their products in plant-based foods can contribute to understanding the mechanism of the chemical reactions^17^, their consequences on human health^16^, and how to develop means to control these reactions during food processing^13^. Therefore, we believe that we can learn from the research on animal protein-based foods and set a starting point to investigate such effects on plant protein-based foods.

Research with animal proteins^19^, such as meat and dairy proteins, has shown that the negative consequences of protein oxidation and Maillard reaction on the nutritional quality of proteins are loss and irreversible modifications of EAAs, as well as impaired protein digestibility^8^. The effect on protein digestibility depends on the level of the oxidation damage: at mild protein oxidation level, partial protein unfolding can facilitate the accessibility of digestive proteases. However, at a severe oxidation level, the resulting irreversible protein aggregation can decrease the susceptibility to the proteases^18,20^. In this recent review, Estevez et al. (2021)^21^ described studies showing that protein carbonylation, an irreversible post-translational modification resulting from oxidative stress, impaired protein digestibility in different animal protein-based foods due to protein aggregation.

As introduced above, the Maillard reaction can also contribute to the reduced nutritional quality of proteins, as the metabolic availability of lysine is reduced due to the formation of glycated lysine^2^. The effect of thermal treatments on glycated lysine formation has been shown in different foods such as soy milk, rapeseed meal, dairy products, and cereal-based foods in which their amount will vary according to the processing and storage conditions^2,22^. Since lysine is an EAA, glycated lysine will negatively impact the protein nutritional quality of foods^23^, especially in cereals in which lysine is a limiting amino acid^7^.

Another effect of food processing on protein nutritional quality is the racemization of l-amino acids to d-amino acids, resulting in a decrease in protein digestibility due to the stereospecificity of the proteases and peptidades^24^. Csapó et al. (2008)^24^ have shown that increasing the extrusion temperature of full-fat soybean increased the amount of d-amino acids, while a loss in the l-amino acids was observed. Among all amino acids, only d-methionine is 100% bioavailable in animals and humans, while others are partially bioavailable in rats, such as d-tryptophan, d-phenylalanine, and d-tyrosine, but the majority are not bioavailable^25^.

Furthermore, protein modifications induced by food processing can have negative consequences on health. For instance, the absorption of oxidized proteins results in elevated levels of reactive oxidation species in the blood and organs that can potentially induce pathogenesis of the body, as shown in experimental animals^18^. Moreover, the decrease in protein digestibility as a result of protein oxidation has been associated with the accumulation of undigested proteins in the colon which induce changes in the composition and abundance of the gut microbiota, resulting in negative changes in its homeostasis^26^.

Therefore, considering that the protein digestibility and some of the EAAs are limiting in plant proteins, attention should be made to identifying modifications that can impair the protein nutritional quality in plant-based foods, to minimize these modifications. For this purpose, we will discuss in the next section the use of more advanced techniques to identify protein modifications on a molecular level.

## Advanced tools to understand in-depth the nutritional quality of plant proteins

Foodomics is an in-depth study of food and nutrition domains through the application and integration of omics technologies to improve consumers’ health. The most common omics technologies used in food and nutrition include transcriptomics, genomics, proteomics, peptidomics, and metabolomics^27^. While transcriptomics and genomics provide information on the transcriptional activity and the genome level^28^, proteomics, peptidomics, and metabolomics provide valuable information about protein and peptide profiles, post-translational modifications, and metabolite profiles during food production and after digestion. Foodomics has been applied in food safety and quality, identification of biomarkers of food intake and diseases, and health effects of foods providing a link between food and human nutrition^28^. Foodomics has also been applied to integrate food processing with human digestion, bioavailability, and health markers in a holistic approach known as Enginomics^29^. In the scenario of plant protein foods, there are only a few studies using foodomics to characterize plant proteins and determine their metabolite profile after food processing or after digestion.

Proteomics can provide information about molecular changes during food processing and aid the understanding of the mechanism of protein modifications, but this approach is still uncharted territory for plant-based foods. Most of the research using proteomics on plant-based foods has been to characterize soy proteins in seeds^30,31^ and to identify allergenic proteins^32,33^. One example of using proteomics to identify Maillard reaction products was shown by Milkovska-Stamenov et al. (2019)^22^ where it was shown that AGEs increased in soy milk products treated under harsh thermal conditions. The identification of AGEs in plant-based products is the first step in understanding under which conditions these products are formed. Follow-up studies are needed to understand how protein modifications can affect plant protein nutritional quality in terms of digestibility and loss of EAAs.

In addition, metabolomics can provide information about changes in metabolites (intermediate or final products of metabolism) during different processing steps and also after digestion^34^. A recent study showed that plant-based meat alternatives with similar nutritional composition as meat products differed by 90% in the metabolite profile compared to beef, using untargeted metabolomics^35^. Plant-based meat had a greater abundance of phenols, tocopherols, and phytosterols than meat. It is not surprising that the metabolite profile differed between the products since both had very different raw materials, despite their macronutrient composition being very similar. Besides the effect of raw materials, the different degrees of processing might also affect the metabolite profile of the foods. Considering the steps in the processing of plant protein-based foods and that protein ingredients might have different degrees of refinement, it is expected that the metabolite profile will differ accordingly. Beleggia et al. (2011)^36^ showed that the metabolite levels changed during pasta production (e.g. amino acids, tocopherols, sugars), from semolina to cooked pasta, depending on the processing conditions. This information helps in understanding which step of the production is most critical, in terms of the degradation of metabolites, providing guidelines about which adjustments should be made to preserve desired metabolites. Therefore, metabolomics is another useful tool that can monitor changes in metabolite profile during food processing from raw material to final food products, and the knowledge obtained can be used to tune processes to produce highly nutritious plant protein-based foods.

Overall, finding an optimal balance between sustainable food production and plant protein nutritional quality is complex and it involves different aspects. Plant proteins have low protein nutritional quality and the use of food processing has been proven to be beneficial because it increases protein digestibility. However, depending on the process conditions applied, this might not be true. Certain processing conditions can induce protein modifications that can hamper protein digestibility and availability of EAAs, depending on the level of the chemical reactions. So far, it is not known if such modifications in plant proteins can harm the nutritional quality of plant-based foods. Therefore, the level of processing will change the nutritional quality of the final food product and most likely it will differ from the raw material and protein ingredients. To access the overall plant protein nutritional quality we recommend the integration of different aspects such as protein modifications induced by food processing and interactions between food matrix components, determination of ANFs and EAAs, and identification of metabolite profile. To obtain in-depth knowledge of plant protein nutritional quality the use of foodomics tools (e.g. proteomics and metabolomics) should be further applied in food science as instruments to monitor changes during and after food processing. In addition, tracking such aspects during the different steps of the processing chain will provide information for improvement on how to adjust processes to obtain plant-based foods with high protein nutritional value, hoping that this will tackle the sustainability paradox.

### Reporting summary

Further information on research design is available in the Nature Research Reporting Summary linked to this article.

## Supplementary information

Reporting summary
